# Editorial: Innate immunity against intracellular bacteria: mechanisms and strategies

**DOI:** 10.3389/fimmu.2024.1396114

**Published:** 2024-03-20

**Authors:** Jingfei Tian, Han Liu, Jingai Che, Lei Song

**Affiliations:** ^1^ Department of Respiratory Medicine, Meihekou Central Hospital, Meihekou, China; ^2^ Department of Respiratory Medicine, Center for Pathogen Biology and Infectious Diseases, The First Hospital of Jilin University, Changchun, China

**Keywords:** autophagy, pyroptosis, pathogen-associated molecular patterns, antimicrobial peptides, phagocytic cells

The battle between the human immune system and intracellular bacteria is a complex and fascinating dance of survival and destruction. Innate immunity, the body’s first line of defense against invading microorganisms, plays a pivotal role in this conflict. This editorial delves into the mechanisms and strategies of innate immunity in combating intracellular bacteria, emphasizing the critical role of the immune system in maintaining human health.

Innate immunity is a non-specific, rapid, and efficient response to infectious agents. It relies on the recognition of conserved molecular patterns common to microorganisms, known as Pathogen-Associated Molecular Patterns (PAMPs) (1). This recognition triggers a cascade of immune responses aimed at eliminating the threat. One of the key strategies of innate immunity against intracellular bacteria is the ability to detect and eliminate infected cells. This process involves the detection of PAMPs by pattern recognition receptors (PRRs) on the surface of phagocytic cells, such as macrophages and dendritic cells (Sankar and Mishra). PRRs recognize bacterial components and initiate signaling cascades that lead to the production of cytokines and other immune mediators. These cytokines then recruit and activate additional immune cells to eliminate the infected cells. Another important strategy is the targeting and destruction of intracellular bacteria by antimicrobial peptides (Duarte-Mata and Salinas-Carmona). These peptides, produced by various immune cells, have the ability to kill bacteria by disrupting their cell membranes or interfering with essential cellular processes. Some antimicrobial peptides even act as signaling molecules to coordinate the immune response (Duarte-Mata and Salinas-Carmona).

Pyroptosis is a recently discovered mechanism by which innate immunity combats intracellular bacteria. This process is characterized by the lysis of infected host cells and the release of intracellular contents, which alerts the immune system to the presence of infection (2). Pyroptosis is initiated by caspase-1 activation in response to PAMPs or damage-associated molecular patterns (DAMPs). Caspase-1 activation leads to the oligomerization of gasdermin D, which forms pores in the cell membrane, causing cell lysis. The release of intracellular bacteria or their components through these pores triggers further immune responses, such as inflammation and recruitment of immune cells. Pyroptosis has been shown to be effective against intracellular bacteria such as *Salmonella enterica* and *Legionella pneumophila* (2).

Autophagy is another recently described mechanism by which innate immunity eliminates intracellular bacteria (3). Autophagy is a process where a cell sequesters cytoplasmic material, including bacteria, into a double-membrane vesicle called an autophagosome. The autophagosome then fuses with lysosomes, where the sequestered material is degraded and eliminated. This process not only removes intracellular bacteria but also provides an antigen presentation platform for macrophages and dendritic cells, enhancing adaptive immune responses. Recent studies have shown that autophagy plays a crucial role in host defense against *Mycobacterium tuberculosis*, *S. enterica*, and *L. pneumophila*, among others (3, 4). The autophagy pathway can be activated by various signals, including PAMPs and cytokines, indicating that it is an integral part of the innate immune response against intracellular bacteria.

However, the battle between intracellular bacteria and the immune system is not a one-sided affair. Intracellular bacteria have evolved various mechanisms to evade or subvert the host immune response (5–7). One such mechanism is the ability to modulate host cell signaling pathways to evade detection by PRRs or to interfere with immune cell activation (8). Other bacteria have developed resistance to antimicrobial peptides or can survive within immune cells, effectively hiding from the immune system (Duarte-Mata and Salinas-Carmona). To counter these evasion strategies, recent research has focused on developing new immunotherapies that can enhance innate immune responses against intracellular bacteria. One such approach involves the use of adjuvants, which are substances that can stimulate the immune response and enhance vaccine potency (9). Other strategies include the development of novel antimicrobial peptides or drugs that can target specific bacterial virulence factors or interfere with their ability to survive within host cells (Duarte-Mata and Salinas-Carmona).

In conclusion, the battle between innate immunity and intracellular bacteria is a dynamic and ongoing arms race. Understanding the mechanisms and strategies of both sides is crucial for developing effective immunotherapies and vaccines against intracellular bacterial infections (Ma et al.; Wan et al.). As we continue to delve into the intricacies of this conflict, we gain valuable insights into how our immune system works and how we can harness its power to combat infectious diseases.

## Author contributions

TJ: Writing – original draft. HL: Writing – original draft. CJ: Writing – review & editing, Writing – original draft. LS: Writing – review & editing, Writing – original draft, Funding acquisition.
